# The TBK1/IKK**ε** inhibitor amlexanox improves dyslipidemia and prevents atherosclerosis

**DOI:** 10.1172/jci.insight.155552

**Published:** 2022-09-08

**Authors:** Peng Zhao, Xiaoli Sun, Zhongji Liao, Hong Yu, Dan Li, Zeyang Shen, Christopher K. Glass, Joseph L. Witztum, Alan R. Saltiel

**Affiliations:** 1 Department of Biochemistry and Structural Biology and; 2 Mays Cancer Center, University of Texas Health Science Center at San Antonio, San Antonio, Texas, USA.; 3 Department of Medicine, University of California, San Diego (UCSD), La Jolla, California, USA.; 4 Department of Pharmacology and; 5 Transplant Center, University of Texas Health Science Center at San Antonio, San Antonio, Texas, USA.; 6 Department of Cellular and Molecular Medicine, School of Medicine;; 7 Department of Bioengineering, Jacobs School of Engineering; and; 8 Department of Pharmacology, School of Medicine, UCSD, La Jolla, California, USA.

**Keywords:** Metabolism, Cardiovascular disease, Cholesterol

## Abstract

Cardiovascular diseases, especially atherosclerosis and its complications, are a leading cause of death. Inhibition of the noncanonical IκB kinases TANK-binding kinase 1 and IKKε with amlexanox restores insulin sensitivity and glucose homeostasis in diabetic mice and human patients. Here we report that amlexanox improves diet-induced hypertriglyceridemia and hypercholesterolemia in Western diet–fed (WD-fed) *Ldlr*^–/–^ mice and protects against atherogenesis. Amlexanox ameliorated dyslipidemia, inflammation, and vascular dysfunction through synergistic actions that involve upregulation of bile acid synthesis to increase cholesterol excretion. Transcriptomic profiling demonstrated an elevated expression of key bile acid synthesis genes. Furthermore, we found that amlexanox attenuated monocytosis, eosinophilia, and vascular dysfunction during WD-induced atherosclerosis. These findings demonstrate the potential of amlexanox as a therapy for hypercholesterolemia and atherosclerosis.

## Introduction

Metabolic diseases have become a worldwide epidemic (1). Atherosclerosis and its complications, including heart attack and stroke, are the leading causes of death (2, 3). The origins of atherosclerosis are complex and multifactorial and are often linked via common underlying mechanisms. For example, hypercholesterolemia and hypertriglyceridemia are frequently associated with chronic inflammation, leading to excessive accumulation of monocyte-derived macrophages in the arterial wall that contributes to the development of atherosclerotic plaques (2, 4–6). Atherosclerosis is currently treated primarily with statins, ezetimibe, and proprotein convertase subtilisin kexin type 9 (PCSK9) inhibitors to decrease plasma cholesterol (7–10). Niacin was also shown to decrease LDL-cholesterol and increase HDL-cholesterol (11–13). However, the use of these agents is not always optimally efficacious and at times is associated with problems. Some individuals cannot tolerate statins, the most widely used agents, due to myopathies and occasionally increased blood glucose and insulin resistance (14–16). Other drugs that reduce triglycerides (fibrates) or decrease bile acid reabsorption (bile acid sequestrants) are not as effective as statins and carry other liabilities (17–19). Although novel PCSK9 inhibitors, alirocumab and evolocumab, have recently been introduced to control cholesterol in patients who do not respond to statins, these drugs are expensive (20–22). Thus, there is a need for new safe and effective drugs to combat atherosclerosis.

Obesity is characterized by low-grade, persistent inflammation in adipose tissue and liver, involving the recruitment and activation of proinflammatory immune cells (23–25). These inflammatory events are characterized by activation of the transcriptional factor NF-κB in both immune cells and metabolically active hepatocytes and adipocytes, linking obesity to both cardiovascular and metabolic disease (26–29). Studies from our laboratory on the NF-κB pathway in adipose tissue and liver from obese mice revealed that both the noncanonical IκB kinases (IKKs), IKKε and TANK-binding kinase 1 (TBK1), are elevated in obesity due to NF-κB activation and further that both proteins play a role in suppressing energy expenditure in the obese state (28, 30). These findings led us to discover amlexanox as a specific inhibitor of both kinases (30). This drug was developed in the mid-1980s to treat asthma and allergic rhinitis (31, 32) and has an excellent record of safety. We demonstrated that amlexanox substantially improved glucose tolerance, fatty liver, and insulin sensitivity; reduced hepatic steatosis in genetically obese and diet-induced obese mice (30, 33, 34); and significantly reduced hemoglobin A1c (HbA1c) levels in a subset of patients with diabetes with high basal levels of systemic inflammation (35). Mechanistic studies revealed that amlexanox reduced expression of proinflammatory cytokine genes *Ccl2* and *Ccl3* and attenuated inflammation (30). Moreover, amlexanox inhibits IKK-induced activation of phosphodiesterase 3B to elevate cAMP levels and p38 phosphorylation in adipocytes and thus increases catecholamine sensitivity and energy expenditure via increased adipose tissue browning and thermogenesis (36, 37). However, it is unknown whether amlexanox could affect other diet-induced metabolic diseases, especially atherosclerosis. In this study, we assessed the effects of amlexanox on Western diet–induced (WD-induced) atherosclerosis in LDL receptor–knockout (*Ldlr*^–/–^) mice. We examined its effects on lipid metabolism, inflammation, and vascular dysfunction and demonstrated that amlexanox systemically ameliorated 3 major pathogenic mechanisms that promote atherogenesis. Given its beneficial effects in obesity, diabetes, and fatty liver diseases, here we demonstrate the potential of amlexanox as a simultaneous treatment for atherosclerosis and other metabolic diseases, including diabetes and fatty liver disease.

## Results

### Amlexanox improves dyslipidemia and protects against atherosclerosis.

**Figure 1:**
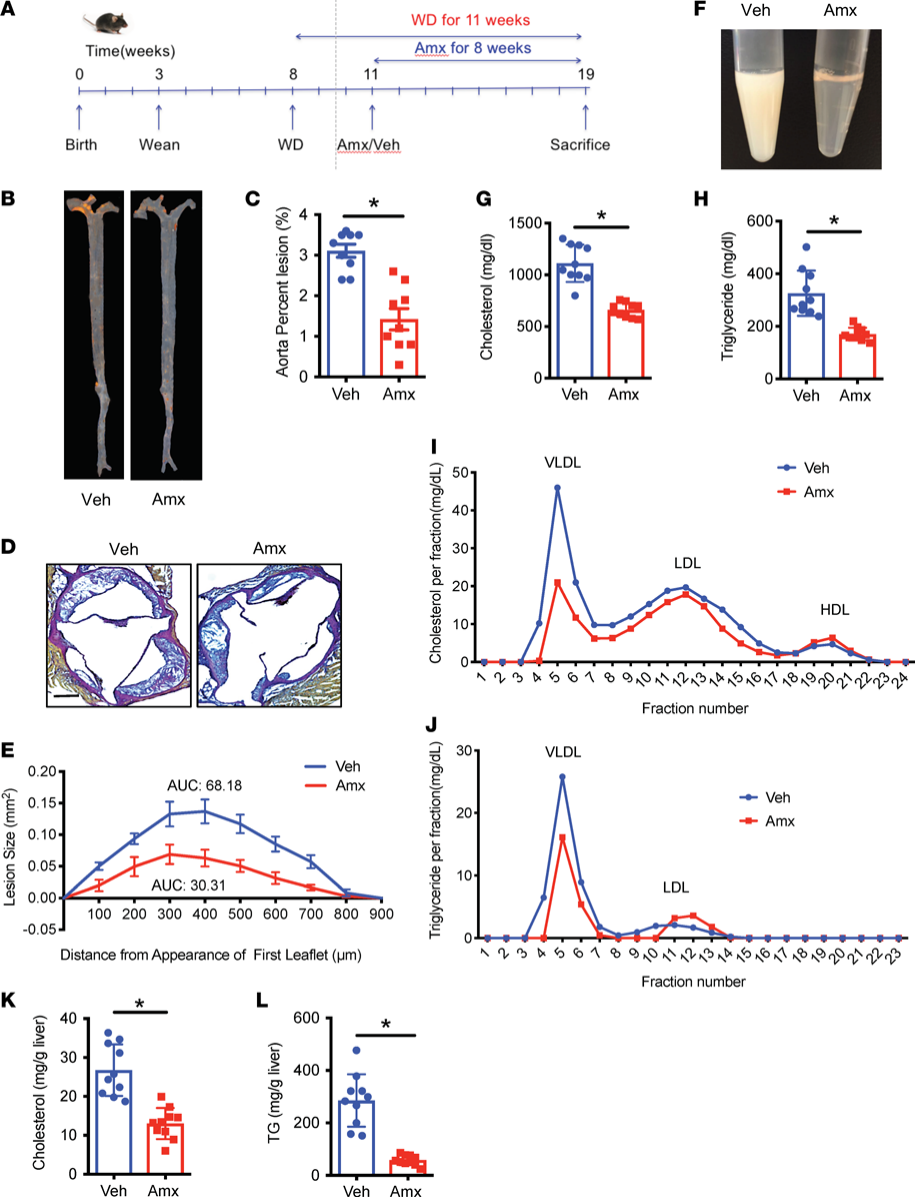
Amlexanox improves dyslipidemia and protects against atherosclerosis. *Ldlr*^–/–^ mice were fed WD for 3 weeks, then orally gavaged with vehicle or amlexanox (25 mg/kg BW) for 8 weeks with the continuation of WD feeding. (**A**) Schematic diagram of experimental design and mouse model. (**B**) En face staining of aorta. (**C**) Quantification of lesion areas in **B**. (**D**) Van Gieson elastic staining of aortic roots. Scale bar, 300 μm. (**E**) Quantification of aortic root staining in **D**. (**F**) Photo of serum from WD-fed *Ldlr^–/–^* mice treated with vehicle or amlexanox. (**G**) Fasting serum cholesterol level. (**H**) Fasting serum triglycerides level. (**I** and **J**) Distribution of plasma cholesterol (**I**) and triglycerides (**J**) by fast performance liquid chromatography (FPLC) in pools of equal aliquots of plasma from vehicle- or amlexanox-treated WD-fed *Ldlr^–/–^* mice (*n* = 5). (**K**) Hepatic cholesterol levels. (**L**) Hepatic triglyceride levels. Mean ± SEM. *, *P* < 0.05, Student’s unpaired *t* test.

Amlexanox is a selective inhibitor of the protein kinases TBK1 and IKKε (30), and its administration to obese rodents or humans improves energy and glucose metabolism (30, 35). To examine whether amlexanox exerts a beneficial effect on WD-induced atherosclerosis, we fed *Ldlr*^–/–^ mice with WD for 3 weeks, then orally gavaged the mice with vehicle or amlexanox for 8 weeks with the continuation of WD feeding (Figure 1A). Consistent with our previous findings, amlexanox improved diet-induced obesity, indicated by significantly reduced body weight and adipose tissue weight in WD-fed mice (Supplemental Figure 1, A–D; supplemental material available online with this article; https://doi.org/10.1172/jci.insight.155552DS1). After 11 weeks of WD feeding, aortas were collected to evaluate lesion development. En face staining demonstrated that amlexanox substantially reduced the area of aortic lesions (Figure 1, B and C). Staining of aortic roots also showed that amlexanox substantially reduced the size of lesions (Figure 1, D and E). Together, these data demonstrated that amlexanox reduced atherogenesis in WD-fed *Ldlr*^-/-^ mice.

Our previous studies demonstrated that amlexanox reduced blood glucose and improved insulin sensitivity in *ob/ob* and HFD-fed mice (30). We thus examined the impact of amlexanox on diet-induced dyslipidemia, including hypertriglyceridemia and hypercholesterolemia. We found that mice gavaged with amlexanox had clear serum, while serum from mice in the vehicle group was milky (Figure 1F), indicating a robust reduction in serum lipid content in response to the drug. Measurement of circulating levels of triglycerides and cholesterol demonstrated that 8-week gavage of amlexanox significantly reduced both triglycerides and cholesterol in WD-fed *Ldlr*^–/–^ mice (Figure 1, G and H). Fast performance liquid chromatography (FPLC) analysis indicated that amlexanox reduced both VLDL-cholesterol and LDL-cholesterol and slightly increased HDL-cholesterol. VLDL-triglycerides were also substantially reduced by amlexanox (Figure 1, I and J). Moreover, amlexanox significantly reduced liver weight (Supplemental Figure 1E), and H&E staining indicated an improvement of hepatic steatosis (Supplemental Figure 1F). Measurement of hepatic lipid content confirmed a dramatic reduction of both triglycerides and cholesterol by amlexanox in WD-fed *Ldlr*^–/–^ mice (Figure 1, K and L). In normal chow diet–fed *Ldlr*^–/–^ mice, amlexanox showed no effects on serum cholesterol and triglycerides (Supplemental Figure 1, G and H). These data demonstrated that amlexanox significantly reduced triglycerides and cholesterol in both blood and liver of WD-fed *Ldlr*^–/–^ mice. We also performed a lipidomics study to specifically assess the impact of amlexanox treatment and demonstrated changes in the distribution of fatty acid species in the serum. Although amlexanox did not affect circulating levels of total fatty acids or saturated fatty acids, it mildly increased the levels of unsaturated fatty acids (Supplemental Figure 2, A–C). Among the saturated fatty acids, amlexanox increased the levels of 17:0, 18:0, 20:0, while reducing 12:0, 14:0, 22:0, 23:0, 24:0, and 26:0 (Supplemental Figure 2D). Among the unsaturated fatty acids, amlexanox upregulated the levels of 18:2, 18:3 N3, 18:3 N6, 20:2, 20:5, 22:4, and 22:5 N6, while downregulating the levels of 16:1, 17:1, 18:1, 20:1, 20:3 N3, 20:4, 22:5 N3, 22:6, and 24:1 (Supplemental Figure 2E). Together, these data demonstrated that amlexanox markedly improved dyslipidemia in WD-fed *Ldlr*^–/–^ mice.

### Amlexanox increases bile acid synthesis and cholesterol excretion.

**Figure 2:**
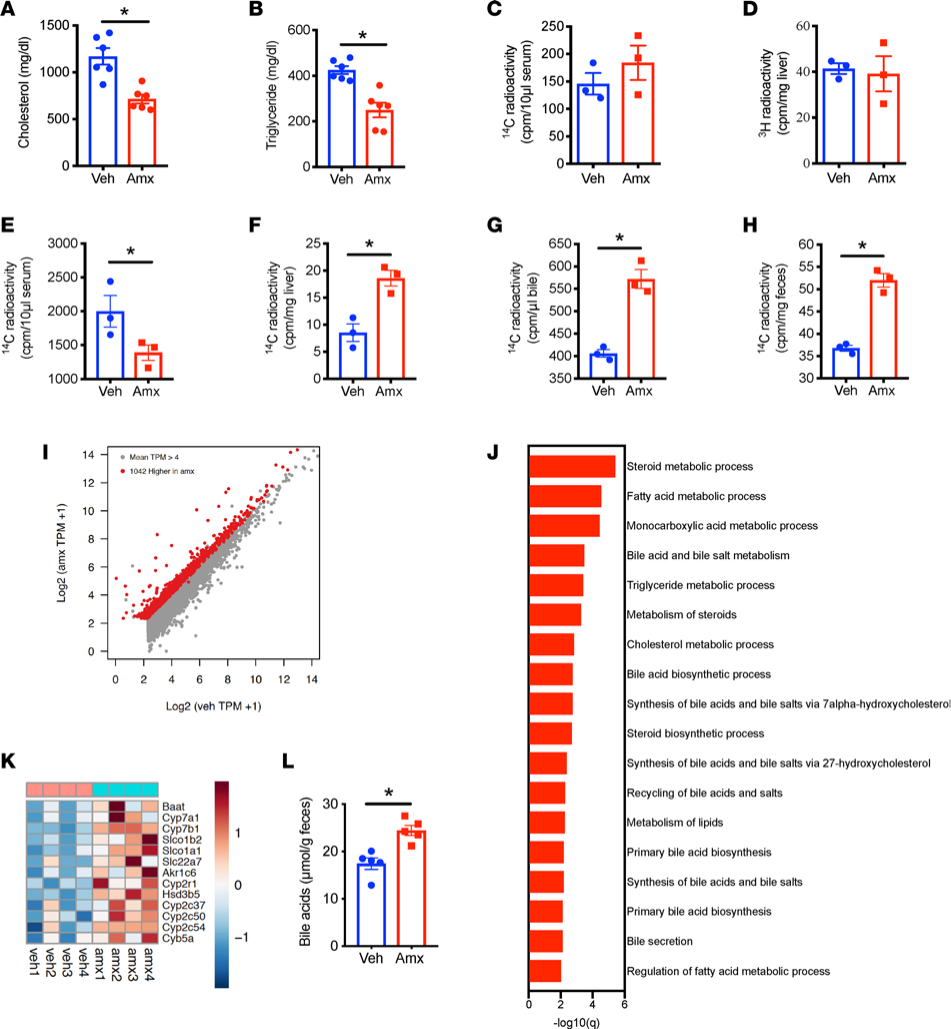
Amlexanox increases bile acid synthesis and cholesterol excretion. (**A**–**E**) Cholesterol metabolism in *Ldlr*^–/–^ mice fed WD for 8 weeks, then orally gavaged with vehicle or amlexanox for 4 weeks with the continuation of WD feeding. (**A**) Fasting serum cholesterol. (**B**) Fasting serum triglycerides. (**C**) Serum ^14^C activity 2 hours after gavage of a mixture of cholesterol and ^14^C-cholesterol. (**D**) ^3^H activity in liver sterol fraction 2 hours after injection of ^3^H-acetate. (**E**–**H**) ^14^C activity in serum (**E**), liver lysate (**F**), bile (**G**), and feces (**H**) 21 hours after gavage of a mixture of cholesterol and ^14^C-cholesterol. (**I**–**K**) Transcriptomic profiling of livers from *Ldlr*^–/–^ mice fed WD for 3 weeks, then orally gavaged with vehicle or amlexanox for 8 weeks with the continuation of WD feeding. (**I**) Scatterplot for RNA-Seq data: 1,042 genes had higher expression levels in Amx. (**J**) Functional annotation associated with genes expressed more highly in amlexanox-treated mice. (**K**) Relative expression values (*Z*-scaled log_2_[TPM+1]) for genes involved in bile acid metabolism. (**L**) Fecal bile acid levels in *Ldlr*^–/–^ mice fed WD for 3 weeks, then orally gavaged with vehicle or amlexanox for 8 weeks with the continuation of WD feeding. Mean ± SEM. *, *P* < 0.05, Student’s unpaired *t* test. TPM, transcript per kilobase million.

Our previous study demonstrated that amlexanox significantly increases energy expenditure to improve obesity and hypertriglyceridemia (30). To elucidate the mechanism by which amlexanox ameliorates hypercholesterolemia in WD-fed *Ldlr*^–/–^ mice, we systematically examined cholesterol metabolism. Mice were fed WD for 8 weeks with 4 additional weeks of feeding along with vehicle or amlexanox administration. The 4 weeks of amlexanox treatment significantly reduced circulating levels of cholesterol and triglycerides in WD-fed *Ldlr*^–/–^ mice (Figure 2, A and B). We assessed the rate of cholesterol absorption by assaying serum ^14^C radioactivity 2 hours after gavage of cold cholesterol mixed with ^14^C-cholesterol. These data demonstrated that amlexanox did not affect cholesterol absorption (Figure 2C). Because liver is the major site of cholesterol synthesis, we also examined hepatic cholesterol synthesis rate in response to amlexanox administration. Mice gavaged with vehicle or amlexanox were injected with ^3^H-acetate, and ^3^H radioactivity in the liver sterol fraction was determined after 2 hours. Our results demonstrated that amlexanox did not affect radioactivity in the liver sterol fraction (Figure 2D), indicating no effect on cholesterol synthesis.

Given that amlexanox does not affect cholesterol absorption or synthesis, we examined whether the drug might control cholesterol excretion to attenuate hypercholesterolemia. WD-fed *Ldlr*^–/–^ mice received vehicle or amlexanox, followed by oral gavage with cold cholesterol mixed with ^14^C-cholesterol. After 21 hours, radioactivity was measured in serum, feces, and liver lysates. Interestingly, radioactivity in serum was significantly reduced in mice gavaged with amlexanox but significantly increased in the liver lysates, bile, and feces, indicating a dramatic increase in cholesterol clearance (Figure 2, E–H).

To understand the mechanism by which amlexanox increases cholesterol excretion, we performed RNA-Seq on liver tissue of vehicle or amlexanox-treated mice. Our data revealed that amlexanox induced profound transcriptional changes in the livers of WD-fed *Ldlr*^–/–^ mice (Figure 2I and Supplemental Figure 3A). Ontological analysis of genes upregulated by amlexanox showed significant enrichment of pathways that mediate bile acid synthesis and metabolism (Figure 2J). A notable example is the higher expression of Cyp7a1, the rate-limiting enzyme of bile acid synthesis (Figure 2K). Amlexanox also upregulated the expression of genes involved in fatty acid metabolism, while downregulating the expression of inflammation genes (Supplemental Figure 3, B and C).

Cholesterol is mainly excreted in the form of bile acids. To ascertain whether amlexanox affects bile acid synthesis and excretion, we measured bile acid levels in the feces of mice gavaged with vehicle or amlexanox. Amlexanox significantly increased the amount of fecal bile acids (Figure 2L). Together, these data suggest that inhibition of the protein kinases TBK1 and IKKε by amlexanox upregulates bile acid production to increase cholesterol excretion and thus ameliorates hypercholesterolemia.

### Amlexanox reduces circulating monocytes and lesion macrophages.

**Figure 3:**
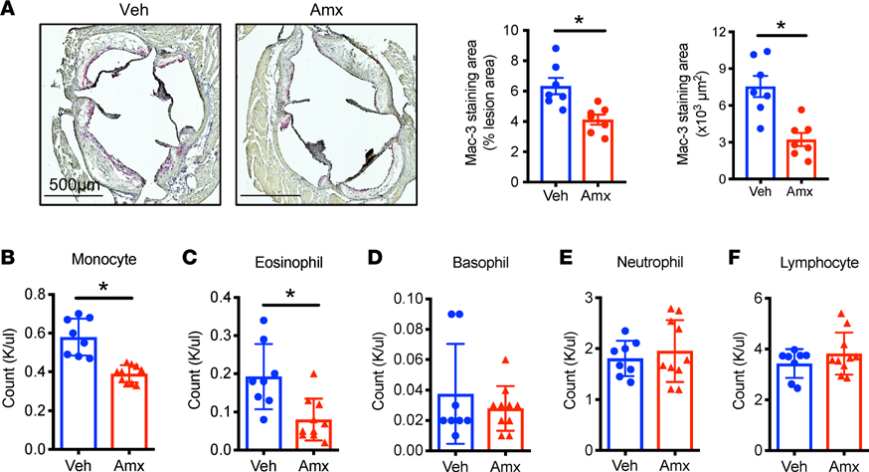
Amlexanox reduces circulating monocytes and lesion macrophages. *Ldlr*^–/–^ mice were fed WD for 3 weeks, then orally gavaged with vehicle or amlexanox for 8 weeks with the continuation of WD feeding. (**A**) Mac-3 staining of aortic roots and quantification. (**B**–**F**) Complete blood count of circulating immune cells: monocytes (**B**), eosinophils (**C**), basophils (**D**), neutrophils (**E**), and lymphocytes (**F**). Mean ± SEM. *, *P* < 0.05, Student’s unpaired *t* test.

Inflammation is a major pathogenic factor for atherosclerosis. Under atherosclerotic conditions, macrophages infiltrate into the blood vessel wall and promote the formation of the necrotic core, which is a defining feature of unstable plaques (38). Staining of plaques with the macrophage marker macrophage antigen-3 (Mac-3) showed a significant attenuation of macrophages in aortic lesions (Figure 3A). To understand the underlying mechanism, we did a complete blood count to evaluate circulating immune cells. Interestingly, we found that amlexanox significantly reduced the numbers of monocytes and eosinophils, while neutrophil, lymphocyte, and basophil numbers were unaffected (Figure 3, B–F). Circulating monocytes are the major source of infiltrated macrophages, and monocytosis has been linked to atherosclerosis (38). These data indicate that amlexanox attenuates monocytosis, which in turn would lead to decreased numbers of monocytes recruited to the artery wall, contributing to the decrease of atherosclerosis. In contrast, amlexanox had no effect on the number of blood monocytes in mice fed normal chow diet (Supplemental Figure 4). We note that amlexanox was originally developed as an asthma treatment (39). Since eosinophilia is a major pathogenic factor in the development of asthma, the anti-eosinophilic effects of the drug might provide insight into the mechanism of amlexanox’s anti-asthma function.

### Amlexanox protects against vascular dysfunction in vitro and in vivo.

**Figure 4:**
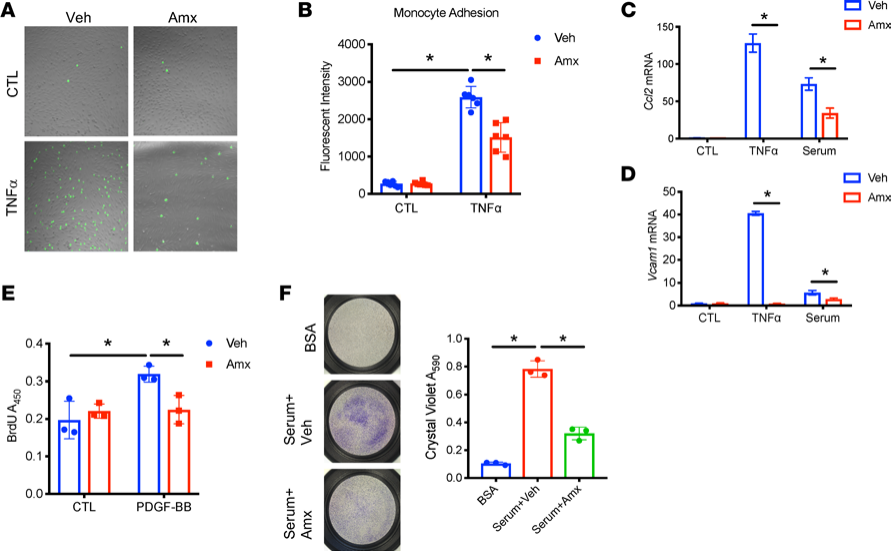
Amlexanox prevents dysfunction of aortic vessel cells. (**A** and **B**) THP-1 monocytes labeled with BCECF were cocultured with HAECs pretreated with vehicle or 100 μM amlexanox, then treated with 10 ng/mL TNF-α for 16 hours. Monocyte adhesion was visualized by confocal microscopy (**A**) or examined by the measurement of fluorescence intensity (**B**). (**C** and **D**) *Ccl2* (**C**) and *Vcam1* (**D**) expression in HAECs treated with 5 nM TNF-α or 10% serum in the presence of vehicle or 100 μM amlexanox. (**E**) BrdU proliferation assay on mouse aortic SMCs pretreated with vehicle or 100 μM amlexanox, then treated with 20 ng/mL PDGF-BB for 24 hours. (**F**) Transwell migration assay of SMCs pretreated with vehicle or 100 μM amlexanox, then treated with serum from WD-fed *Ldlr*^–/–^ mice. Mean ± SD. *, *P* < 0.05, Student’s unpaired *t* test.

Vascular dysfunction is a major contributor to the pathogenesis of atherosclerosis (40–43). Endothelial cells that are dysregulated in atherosclerosis trigger monocyte adhesion and thus promote macrophage infiltration into the aortic vessel (3, 4, 41–43). Increased proliferation and migration of smooth muscle cells (SMCs) is also an indispensable component of plaque formation (44, 45). To understand whether amlexanox affects monocyte-endothelial cell adhesion, we labeled THP-1 monocytes with the radiometric pH indicator 2′,7′-Bis-(2-Carboxyethyl)-5-(and-6)-Carboxyfluorescein (BCECF) and treated human aortic endothelial cells (HAECs) with vehicle or TNF-α. After removal of BCECF and TNF-α, monocytes were incubated with HAECs. Nonadherent monocytes were washed away, and monocytes adhering to endothelial cells were visualized by fluorescence microscopy. As expected, TNF-α increased monocyte-endothelial cell adhesion (Figure 4, A and B). Gene expression analysis on HAECs demonstrated that TNF-α and serum from WD-fed *Ldlr*^–/–^ mice induced the expression of the inflammatory markers *Ccl2* and *Vcam1* in endothelial cells. These effects were significantly attenuated by the pretreatment of cells with amlexanox (Figure 4, C and D). The data suggested that amlexanox downregulated the TNF-α–dependent expression of *Ccl2* and *Vcam1* in endothelial cells to attenuate monocyte adhesion.

To elucidate the site of amlexanox action, we determined whether amlexanox affects the function of SMCs to prevent plaque formation, by performing a BrdU labeling assay on mouse vascular smooth muscle cells (MOVAS). Our data demonstrated that platelet-derived growth factor BB (PDGF-BB) significantly induced SMC proliferation. This effect was substantially inhibited by the pretreatment of cells with amlexanox (Figure 4E). We also utilized a Transwell cell migration assay to study amlexanox’s effect on SMC migration. Amlexanox significantly reduced the migration of SMCs induced by the addition of serum from WD-fed *Ldlr*^–/–^ mice (Figure 4F). Together, these findings demonstrate that amlexanox attenuates the proliferation and migration of SMCs, which could contribute to the amelioration of atherogenesis.

**Figure 5:**
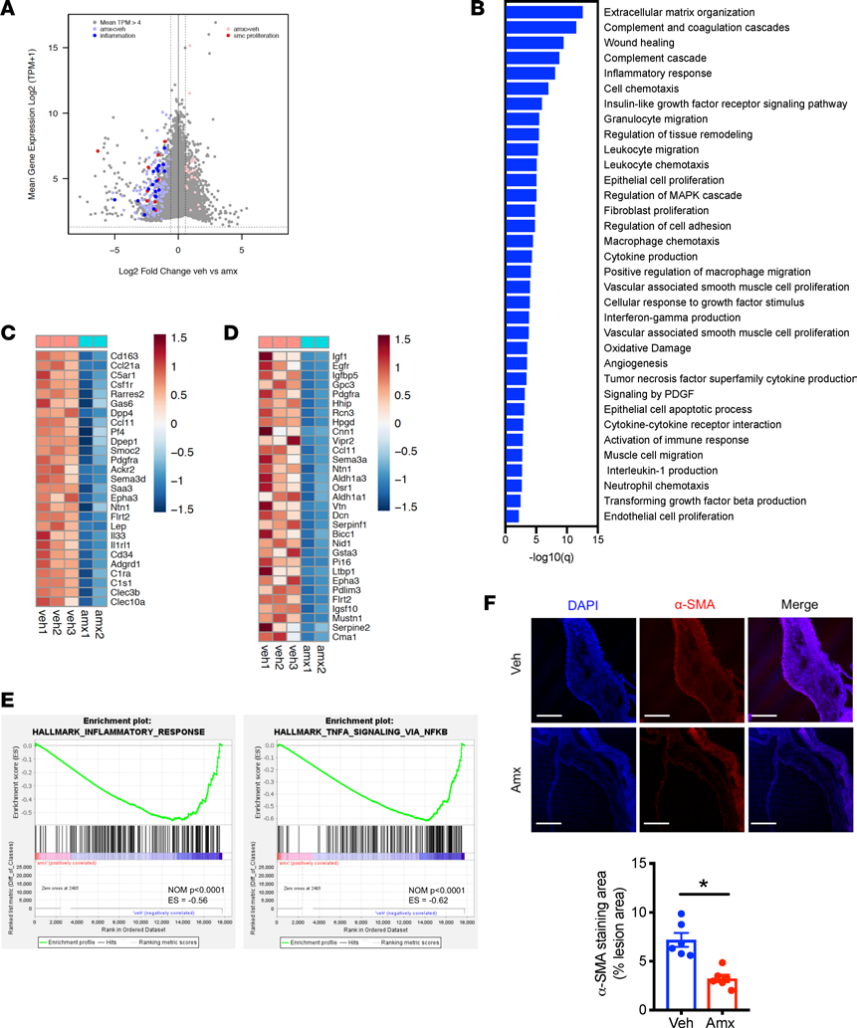
Amlexanox affects aortic transcriptome during atherogenesis. (**A**–**E**) Transcriptomic profiling of aortas from *Ldlr*^–/–^ mice fed WD for 3 weeks, then orally gavaged with vehicle or amlexanox for 8 weeks with the continuation of WD feeding. (**A**) MA plot of mRNA expression. (**B**) Functional annotation associated with genes expressed lower in amlexanox-treated mice. (**C** and **D**) Relative expression values (*Z*-scaled log_2_[TPM+1]) for genes involved in inflammation (**C**) or SMC proliferation and migration (**D**). (**E**) Gene set enrichment analysis of differentially expressed transcripts related to inflammatory response and TNF-α signaling in aortas of vehicle- or amlexanox-treated WD-fed *Ldlr^–/–^* mice. (**F**) Staining for α-SMA of aortic roots and quantification. Scale bar, 200 μm. *Ldlr*^–/–^ mice were fed WD for 3 weeks, then orally gavaged with vehicle or amlexanox for 8 weeks with the continuation of WD feeding. Mean ± SEM. *, *P* < 0.05, Student’s unpaired *t* test.

To elucidate the underlying mechanism by which amlexanox prevents cardiovascular dysfunction to protect against atherosclerosis, we dissected aortas from WD-fed *Ldlr*^–/–^ mice gavaged with vehicle or amlexanox and performed RNA-Seq analysis on whole aortas. We observed 157 genes with levels of expression in aortas of amlexanox-treated mice that were at least 1.5-fold less than vehicle-treated aortas at an FDR of 0.05 and TPM greater than 16 (Figure 5A and Supplemental Figure 5A). Ontological analysis of genes downregulated by amlexanox demonstrated significant functional enrichment of atherogenic related categories, such as inflammatory response, cell chemotaxis, SMC proliferation, and cell migration (Figure 5B). The expression of genes involved in inflammation and SMC proliferation and migration is shown in Figure 5, C and D. Gene set enrichment analysis for differentially expressed transcripts in aortas revealed a significant downregulation of the inflammatory response, TNF-α signaling, TGF signaling, and apoptotic pathways by amlexanox (Figure 5E and Supplemental Figure 5B). Staining for α–smooth muscle actin (α-SMA) showed a significant reduction of SMCs within the aortic lesions (Figure 5F), confirming that amlexanox attenuated SMC migration into plaques.

## Discussion

Atherosclerosis and its complications, like heart attack and stroke, are the leading causes of death in modern society (2, 3). Atherogenesis results from hypercholesterolemia (46, 47), systemic chronic inflammation (3, 48, 49), and aortic cell dysfunctions (40–43, 50), conditions that often appear together in patients with cardiovascular disease. In this study, we found that amlexanox improved all 3 aspects of this syndrome to prevent atherosclerosis. Hypercholesterolemia, especially high blood concentrations of VLDL- and LDL-cholesterol, is essential for the development of aortic plaques (46). We showed that amlexanox significantly improved dyslipidemia and reduced both VLDL-cholesterol and LDL-cholesterol in WD-fed *Ldlr*^–/–^ mice. Chronic inflammation is characterized by penetration of monocytes into aortic vessels, and monocyte-derived macrophages form the necrotic core of atherosclerotic lesions (3, 51). Infiltration of monocytes/macrophages modulates the development of lesions and affects their stability through cytokine secretion and crosstalk with artery wall cells (5). Oral gavage of amlexanox substantially reduced circulating monocytes as well as macrophages in aortic lesions. Additionally, endothelial cell dysfunction resulted in an upregulation of proinflammatory cytokines and adhesion molecules, which further increase monocyte adhesion (4). The phenotypic switch of SMCs from “contractile” to “synthetic” increases the proliferation of these cells (44). Increased expression of MMPs in aortic vessels cells results in ECM remodeling, which promotes SMC migration into lesions (45). Our study demonstrated that amlexanox not only attenuates monocyte-endothelial adhesion but also reduces SMC proliferation and migration to ameliorate the dysfunction of aortic vessel cells. Taken together, amlexanox improves all 3 aspects of this syndrome to protect against atherogenesis.

To understand the mechanism by which amlexanox improves these pathogenic aspects during atherogenesis, we profiled the transcriptome in liver and aorta. Bioinformatic analyses indicated that amlexanox significantly increased the expression of genes mediating bile acid synthesis in the liver, while attenuating the expression of inflammatory genes and the genes involving SMC proliferation and migration. Our previous study demonstrated that amlexanox inhibits IKKε/TBK1 to attenuate inflammation (30, 35). Here, we showed that amlexanox substantially reduced the expression of inflammation genes in the liver. Given that inflammation, especially via the production of TNF-α, could repress the expression of *Cyp7a1* (52, 53), we speculate that the improvement of systemic inflammation in WD-fed *Ldlr*^–/–^ mice is responsible for the increased expression of bile acid synthesis genes. A recent study showed that amlexanox inhibits IKKε/TBK1 to repress TGF-β production (54). Consistent with this work, we found that amlexanox downregulated TGF-β signaling in aortas, which could result in the attenuation of SMC proliferation and migration.

While current anti-atherosclerosis drugs are effective, their use is limited in certain populations. Most patients with metabolic syndrome present with hypercholesterolemia, hyperglycemia, and insulin resistance. Although the use of statins in these populations reduces the risk of cardiovascular disease (CVD), their use may be associated with the risk of a drug-induced increase in blood glucose, potentially exaggerating diabetes in certain patients (14–16). Therefore, there is a need for new anti-atherosclerotic medications for a subgroup of patients with metabolic syndrome. Our previous studies showed that amlexanox significantly improves insulin sensitivity and glucose metabolism, while reducing hepatic steatosis in obese mice (30, 33, 34). A proof-of-concept clinical study also demonstrated that 12 weeks of amlexanox treatment was safe, and significantly decreased HbA1c levels, an effect that was more pronounced in a subset of patients with high systemic inflammation at baseline (35). The profound effects of the drug prompted us to evaluate amlexanox in a mouse model of atherosclerosis. Our findings demonstrate that amlexanox substantially attenuated diet-induced hypercholesterolemia and reduced aortic lesion area. Mechanistic studies suggest that amlexanox increases cholesterol excretion, prevents monocytosis, and attenuates aortic vessel cell dysfunction, indicating that amlexanox may represent a novel approach to the treatment of hypercholesterolemia and atherosclerosis in statin-resistant or metabolic syndrome patients with hyperglycemia, dyslipidemia, and fatty liver diseases. More importantly, these data illustrate a potentially important role for the amlexanox targets, IKKε and TBK1, as a driver of CVD.

## Methods

### Mice.

Mice were used in accordance with the *Guide for Care and Use of Laboratory Animals* of the NIH (National Academies Press, 2011). Mice were housed in a specific pathogen–free facility with a 12-hour light/12-hour dark cycle and given free access to food and water, except for the fasting period. At 8 weeks of age, 18 to 20 g male *Ldlr*^−/−^ mice (The Jackson Laboratory, 002207) were matched for age, body weight, and total cholesterol and placed on a WD (Research Diets, D12079Bi). Mice were orally gavaged 25 mg/kg amlexanox (Abcam, catalog ab142825) or vehicle every other day with the continuation of WD feeding. During the feeding, mice were weighed monthly. At sacrifice, tissues were weighed. Blood samples were collected. Complete blood count was done by the hematology core at UCSD. Liver H&E staining was carried out by the histology core at UCSD. Stained tissue was visualized with NanoZoomer Slide Scanner (Hamamatsu) at UCSD School of Medicine Microscopy Core. Lipidomic study was performed at the lipidomics core at UCSD.

### Atherosclerotic plaque analysis.

The aortas were dissected under a microscope, fixed in 4% formalin-sucrose, opened, flattened, pinned, and stained with Sudan IV, and images of the aortas were captured and quantified by analysis of the entire en face aorta as previously described (55). Aortic root cross-sectional lesion areas were quantified using serial cross sections taken at 100 μm intervals between 100 μm and 900 μm beginning with the first appearance of the first leaflet of the aortic valve until the last leaflet. Mean lesion size at each 100 μm section in each animal was determined by computer-assisted morphometry on serial 10 μm paraffin sections. Modified van Gieson elastic stain was used to enhance the contrast between the intima and surrounding tissue. Cross-sectional plaque area and plaque morphology were evaluated blindly. The results are presented as mean of all values for each interval plotted versus the distance from the first leaflet, and the overall extent of aortic root lesions was determined by AUC analysis of all serial sections in each group. Mac-3 staining on aortic roots was performed using anti–Mac-3 antibody (Santa Cruz Biotechnology, catalog sc-20004).

### Cholesterol metabolism.

WD-fed *Ldlr*^−/−^ mice were fasted overnight, then intraperitoneally injected with 10 mCi/kg ^3^H-acetate. After 2 hours, ^3^H activity in liver sterol fraction was measured to indicate cholesterol synthesis rate. WD-fed *Ldlr*^−/−^ mice were fasted overnight, then orally gavaged a mixture of 2 mg/kg cholesterol and 20 μCi/kg ^14^C-cholesterol. After 2 hours, serum ^14^C activity was examined as an indication of cholesterol absorption rate. After 21 hours, ^14^C activities in serum, liver lysate, bile, and feces were measured to examine cholesterol excretion.

### Lipid measurement.

Blood/tissue triglyceride and cholesterol levels were determined using the Triglyceride Quantification Colorimetric/Fluorometric Kit (BioVision, Abcam, catalog K622) and Total Cholesterol and Cholesterol Ester Colorimetric/Fluorometric Kit (BioVision, Abcam, catalog K603) according to the manufacturer’s instructions. All values were analyzed from 12-hour-fasted mice.

Lipoprotein profiling was performed on terminal blood samples from a pool of 5 mice using FPLC equipped with a Superose 6 column (GE Healthcare), and total cholesterol and triglyceride levels in each fraction were determined as described above.

### Fecal bile acid measurement.

Feces were weighed out. Bile acids were extracted using 90% ethanol with 0.1N NaOH. Bile acids were determined using Mouse Total Bile Acids Assay Kit (Crystalchem, catalog 80471) according to the manufacturer’s instructions.

### Gene expression analysis.

Analysis of gene expression was performed as previously described (56, 57). Tissues were homogenized in TRIzol reagent (Life Technologies). RNA was isolated with PureLink RNA Mini Kit (Life Technologies). A total of 1 μg of purified RNA was used for reverse transcription PCR to generate cDNA using High-Capacity cDNA Reverse Transcription Kit (Applied Biosystems). ΔΔCt real-time PCR with Power SYBR Green PCR Master Mix (Life Technologies) and QuantStudio 5 Real-Time PCR System were used to analyze cDNA. *Ppia* (cyclophilin A) was used as endogenous control. The following primers were used for quantitative PCR: *Ccl2* forward: CAGCCAGATGCAATCAATGCC; reverse: TGGAATCCTGAACCCACTTCT. *Ppia* forward: GGCAAATGCTGGACCCAACACA; reverse: TGCTGGTCTTGCCATTCCTGGA. *Vcam1* forward: CCGGATTGCTGCTCAGATTGGA; reverse: AGCGTGGAATTGGTCCCCTCA.

### Cell migration assay.

MOVAS (ATCC, catalog CRL-2797) were cultured within the Transwells. Cells were treated with PDGF-BB or serum in the presence of vehicle or amlexanox for 24 hours. MOVAS that migrated to the lower side of Transwells were stained with 0.1% Crystal Violet (Fisher Chemical) and visualized by bright-field microscopy (Keyence). Crystal Violet was eluted by 10% acetic acid. Absorbance was measured at 590 nm for quantification.

### Cell adhesion assay.

HAECs (ATCC, catalog PCS-100-011) were treated with TNF-α or serum for 10 hours in the presence of vehicle or 100 μM amlexanox. THP-1 cells (ATCC) were incubated with 5 μM BCECF (AM) for 30 minutes. Both cell lines were washed with PBS 3 times. THP-1 cells were transferred and cocultured with HAECs for 30 minutes. Nonadherent monocytes were washed away. Monocytes that adhered to endothelial cells were visualized by florescence microscopy (ZEISS).

### BrdU cell proliferation assay.

MOVAS were cultured in a 24-well cell culture plate and treated with PDGF-BB in the presence of vehicle or 100 μM amlexanox for 24 hours. Cell proliferation rate was determined using BrdU Cell Proliferation Assay Kit (BioVision, Abcam, catalog K306) according to the manufacturer’s instructions.

### RNA-Seq library preparation.

Total RNA was isolated from mouse livers, homogenized with TRIzol reagent, and purified using Quick RNA mini prep columns (Zymo) and RNase-free DNase digestion according to the manufacturer’s instructions (Life Technologies). RNA quality was assessed by an Agilent 2100 Bioanalyzer. Sequencing libraries were prepared in biological replicates from poly(A)-enriched mRNA. RNA-Seq libraries were prepared from poly(A)-enriched mRNA as previously described (58). The total RNA-Seq library was prepared by UCSD Institute for Genomic Medicine using Illumina Total RNA prep kit. Libraries were quantified using a Qubit dsDNA HS Assay Kit (Thermo Fisher Scientific) and sequenced on a HiSeq 4000 (Illumina) according to the manufacturer’s instructions.

### RNA-Seq analysis.

RNA-Seq analysis was conducted as previously described (59, 60). FASTQ files from sequencing experiments were mapped to the mouse mm10 genome. STAR with default parameters was used to map RNA-Seq experiments (61). To compare differential gene expression between indicated groups, HOMER’s analyzeRepeats with the option rna and the parameters -condenseGenes, -noadj, and -count exons was used on 2 or 3 replicates per condition (62). Each sequencing experiment was normalized to a total of 107 uniquely mapped tags by adjusting the number of tags at each position in the genome to the correct fractional amount given the total tags mapped. Sequence experiments were visualized by preparing custom tracks for the UCSC Genome Browser. Differential gene expression was assessed with DESeq2 using HOMER’s getDiffExpression.pl with the parameters -*P*-adj 0.05 and -log_2_ fold 0.585 (for 1.5-fold differently expressed genes) (63). For all genes the TPM values were plotted and colored according to fold change. For various ontology analyses, either HOMER or Metascape was used (64). The accession number for the transcriptomic data reported in this paper is Gene Expression Omnibus GSE209621.

### Statistics.

All data in animal studies are shown as mean ± SEM, while data from in vitro studies are shown as mean ± SD. Replicates are indicated in figure legends. *N* represents the number of experimental replicates. An *f* test was performed to determine the equality of variance. When comparing 2 groups, statistical analysis was performed using a 2-tailed Student’s *t* test, except when the *f* test suggested that variances were statistically different. For analysis of more than 2 groups, we used ANOVA to determine equality of variance. Comparisons between groups were performed with Tukey-Kramer post hoc analysis. For all tests, *P* < 0.05 was considered statistically significant.

### Study approval.

The animal studies were approved by the Institutional Animal Care and Use Committees of UCSD and University of Texas Health Science Center at San Antonio.

## Author contributions

PZ and XS conceptualized and designed the study. ARS and JLW supervised the study. PZ, XS, ZL, and HY performed experiments. PZ, XS, ZL, DL, and ZS analyzed data. CKG provided support on atherosclerosis studies and next-generation sequencing. PZ and XS wrote the manuscript. ARS and JLW edited the manuscript.

## Supplementary Material

Supplemental data

## Version 1 08/02/2022

In-Press Preview

## Version 2 09/08/2022

Electronic publication
