# Supplementary material for: The TBK1/IKKε inhibitor amlexanox improves dyslipidemia and prevents atherosclerosis
Source: JCI Insight. 2022 Sep 8;7(17):e155552. doi: 10.1172/jci.insight.155552 (PMC9536260; doi:10.1172/jci.insight.155552)
Supplement: Supplemental data [file jciinsight-7-155552-s018.pdf]

Supplementary Fig. 1

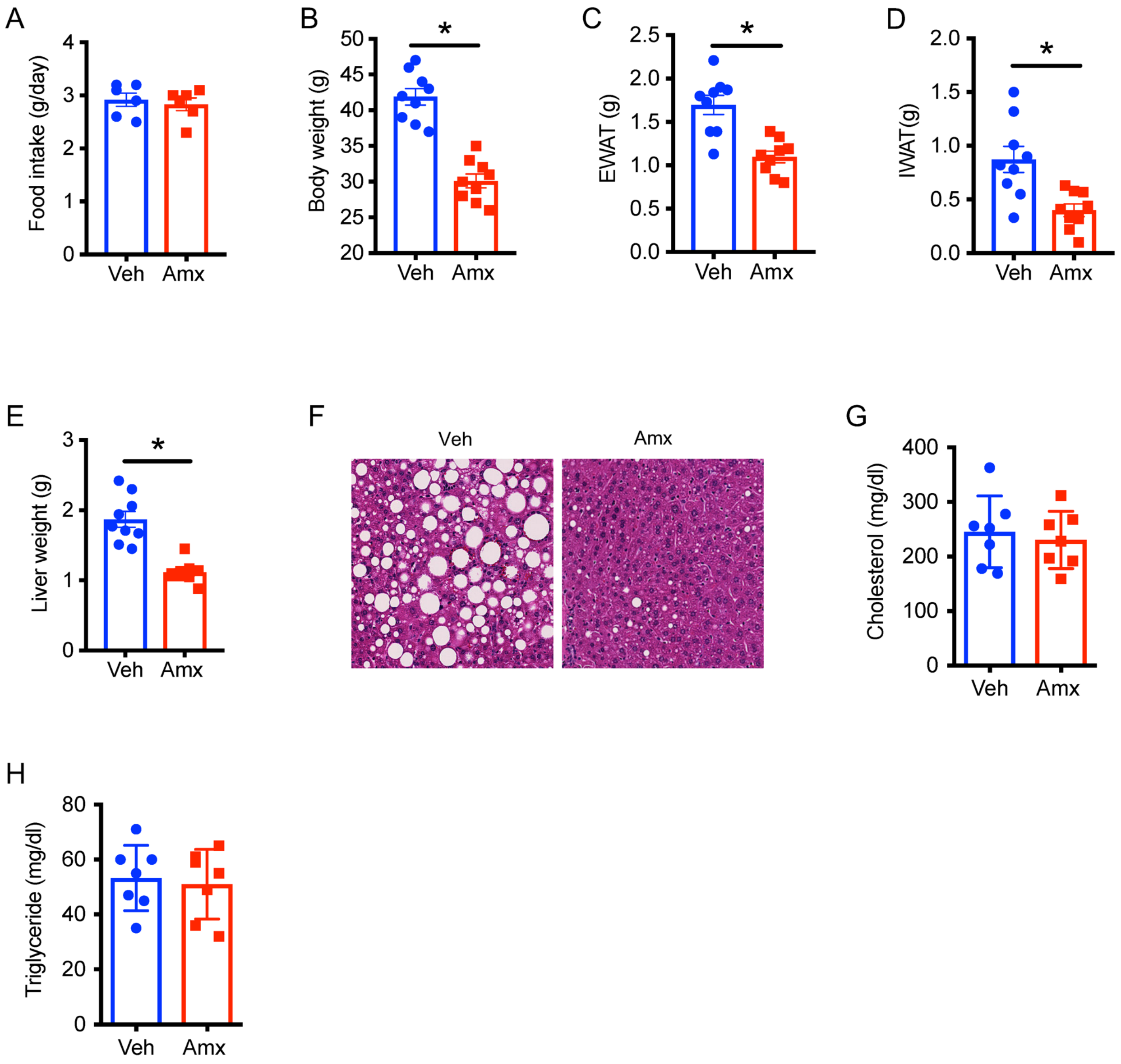

Supplementary Figure 1. Amlexanox reduces body weight and fat mass in mice fed with Western diet. A-F. *Ldlr*$^{-/-}$ mice were fed WD for 3 weeks, then orally gavaged with vehicle or amlexanox for 8 weeks with the continuation of WD feeding. **A.** Food intake. **B.** Body weight. **C.** Weight of epididymal white adipose tissue. **D.** Weight of inguinal white adipose tissues. **E.** Liver weight. **F.** H&E staining of liver sections. **G-H.** *Ldlr*$^{-/-}$ mice were fed normal chow diet for 3 weeks, then orally gavaged with vehicle or amlexanox for 8 weeks with the continuation of chow diet feeding. **G.** Fasting serum cholesterol. **H.** Fasting serum triglyceride. Mean ± SEM. *, P<0.05, Student's unpaired t test.

Supplementary Fig. 2

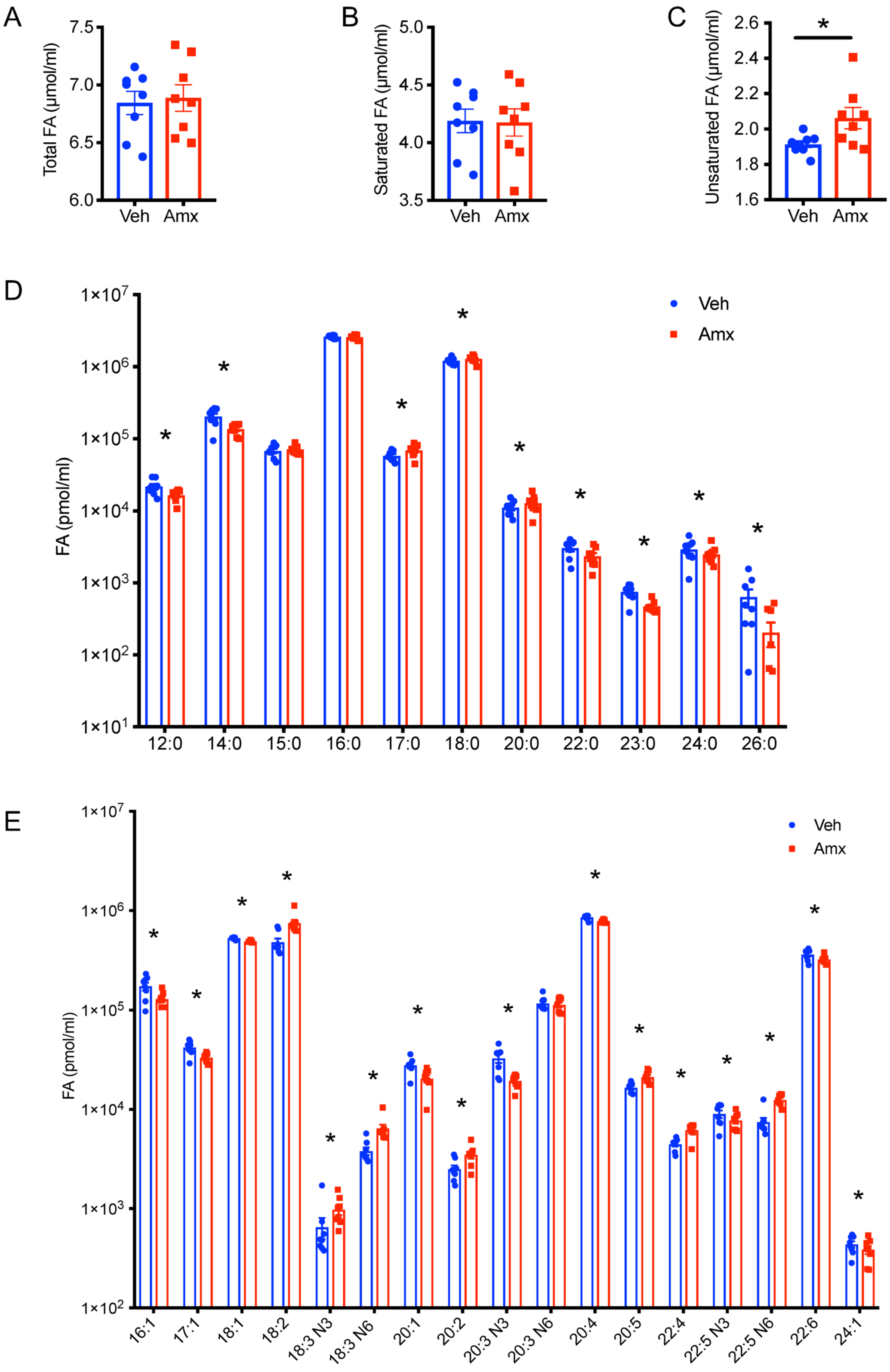

Supplementary Figure 2. The effects of amlexanox on circulating levels of total fatty acids. Lipidomic profiling of plasma from fasted *Ldlr*$^{-/-}$ mice fed WD for 3 weeks, then orally gavaged with vehicle or amlexanox for 8 weeks with the continuation of WD feeding. **A.** Total fatty acids. **B.** Saturated fatty acids. **C.** Unsaturated fatty acids. **D.** Species of saturated fatty acids. **E.** Species of unsaturated fatty acids. Mean ± SEM. *, $P<0.05$, Student's unpaired *t* test.

Supplementary Fig. 3

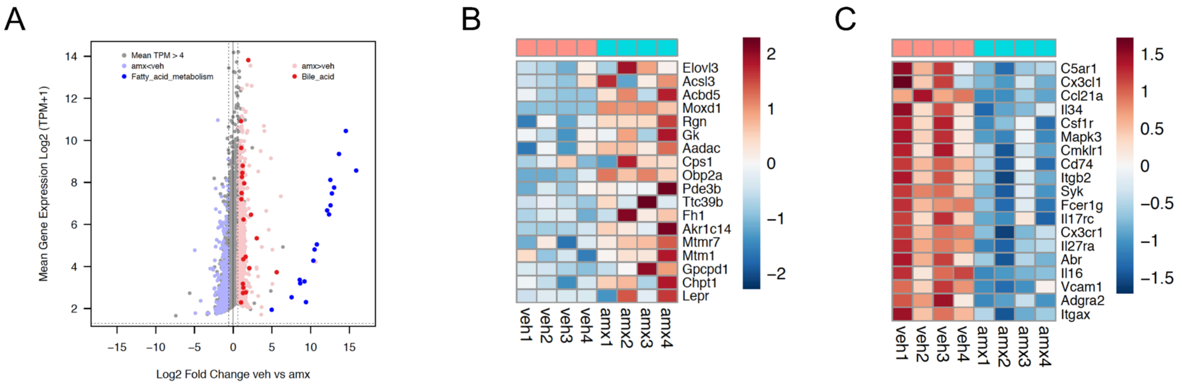

Supplementary Figure 3. Amlexanox affects hepatic transcriptome. Transcriptomic profiling of livers from *Ldlr*$^{-/-}$ mice fed WD for 3 weeks, then orally gavaged with vehicle or amlexanox for 8 weeks with the continuation of WD feeding. **A.** MA plot of RNA-seq data. **B, C.** Relative expression values (Z-scaled $\log_2$(TPM+1)) for genes involved in fatty acid metabolism (B) or inflammation (C).

Supplementary Fig. 4

A

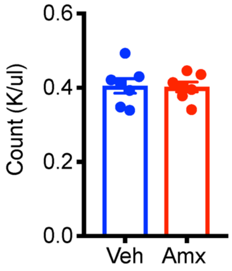

Supplementary Figure 4. Amlexanox does not affect the number of circulating monocytes in mice fed chow diet. Circulating monocytes in *Ldlr*$^{-/-}$ mice were fed normal chow diet for 3 weeks, then orally gavaged with vehicle or amlexanox for 8 weeks with the continuation of feeding.

Supplementary Fig. 5

A

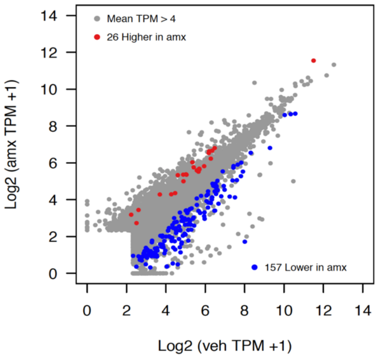

B

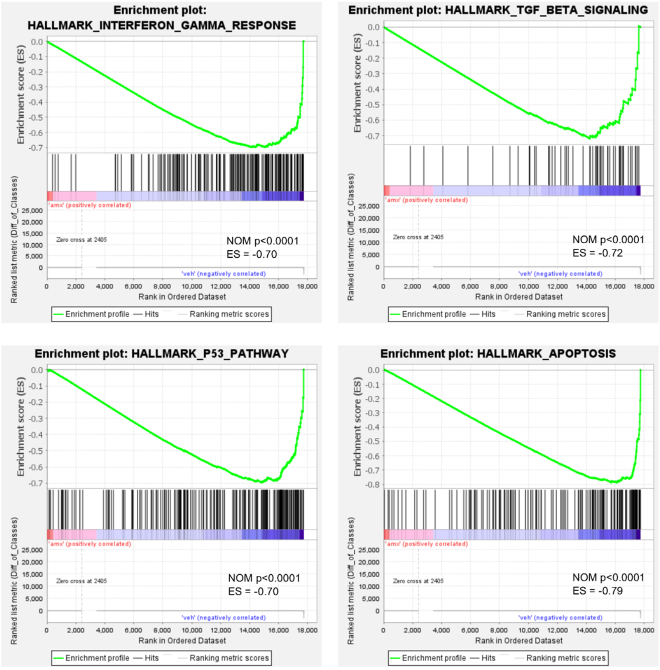

Supplementary Figure 5. Amlexanox affects transcriptome of aortic vessel. Transcriptomic profiling of aorta from *Ldlr*$^{-/-}$ mice fed WD for 3 weeks, then orally gavaged with vehicle or amlexanox for 8 weeks with the continuation of WD feeding. **A.** Scatterplot of RNA-seq data. **B.** Gene Set Enrichment Analysis of differentially expressed transcripts related to interferon gamma response, TGFβ signaling, P53 pathway and apoptosis in aorta of vehicle or amlexanox treated WD-fed *Ldlr*$^{-/-}$ mice.
